# Analysis and comparison of anti-RBD neutralizing antibodies from AZD-1222, Sputnik V, Sinopharm and Covaxin vaccines and its relationship with gender among health care workers

**DOI:** 10.1186/s12979-022-00303-x

**Published:** 2022-10-22

**Authors:** Hamed Zare, Hadis Rezapour, Alireza Fereidouni, Saboura Nikpour, Sara Mahmoudzadeh, Simon G Royce, Mohammad Fereidouni

**Affiliations:** 1 Pharmaceutical Sciences and Cosmetic Products Research Center, Kerman University of Medical Sciences, Kerman, Iran; 2 Student Research Committee , Birjand University of Medical Sciences, Birjand, Iran; 3 Cellular and Molecular Research Center, Birjand University of Medical Sciences, Birjand, Iran; 4 Department of Pharmacology, Monash University, Clayton, Australia

**Keywords:** Vaccine, COVID-19, Sputnik V, AZD-1222, Sinopharm, Covaxin

## Abstract

**Background:**

Vaccine efficiency has a significant role in the public perception of vaccination. The current study was designed to evaluate the efficacy of COVID-19 vaccines (AZD-1222, Sputnik-V, Sinopharm, and Covaxin) and the effect of gender on vaccine efficacy. We evaluated the efficacy of these vaccines among 214 health care employees in Iran. Blood samples were taken from all participants on day 0 and 14 days after the second dose. Humoral responses were evaluated by the PT-SARS-CoV-2-Neutralizing-Ab-96.

**Results:**

The frequency of immunized individuals in the Sputnik V and AZD-1222 groups was 91% and 86%, respectively. This rate was 61% and 67% for Sinopharm and Covaxin vaccines. A comparison of the results obtained from the effectiveness of the vaccines between female and male groups did not demonstrate a significant difference.

**Conclusion:**

According to the results, Sputnik V and AZD-1222 vaccines were more effective than Sinopharm and Covaxin vaccines. Moreover, the effectiveness of these vaccines is not related to gender.

**Supplementary Information:**

The online version contains supplementary material available at 10.1186/s12979-022-00303-x.

## Introduction

SARS-CoV-2 was first recognized as a COVID-19 agent two years ago (December 2019) [1–3]. SARS-CoV-2 has infected more than 595 million individuals worldwide (Aug 2022) and has been responsible for more than 6.45 million deaths [4]. SARS-CoV-2 infection usually causes a wide range of symptoms in people, which can range from mild symptoms to severe manifestations and even death. Also, people who have survived severe infections suffer from post-COVID-19 syndrome such as fatigue, shortness of breath, muscle pains, etc. [5–7]. In addition, convalescent patients may develop other infections (bacterial, viral, and fungal), cardiovascular problems, and other psychological issues [6–8]. Due to the health and economic pressures of the COVID-19 epidemic, vaccination is able to reduce this burden by decreasing the mortality rate of SARS-CoV-2 infection [9, 10].

According to the World Health Organization, more than 137 candidates are currently undergoing clinical development, of which a small number are licensed and approved [11]. The characteristics of an ideal vaccine are: effectiveness after one or two doses of vaccination; protection of target populations such as the elderly and people with underlying disease; efficacy and protection for at least 6 months; and reduce further transmission of the virus to others [5, 12].

AZD-1222 (Oxford/AstraZeneca), BBIBP-CorV (Sinopharm), Sputnik V (Gamaleya Research Institute) and Covaxin (Bharat Biotech) vaccines were investigated in the current study (February to March 2021).

AZD-1222 and Sputnik V are based on a non-replicating adenoviral vector platform. This platform is based on adenoviruses, which are deactivated by removing the E1B and E1A genes, and was established in 1972 [13, 14]. The spike antigen cDNA is inserted into a non-replicating adenoviral vector, and then these vaccines provide the cDNA of the spike protein to infected cells, which leads to spike protein expression in host cells [15]. AZD-1222 and Sputnik V elevate both humoral and cellular immunity [15]. According to the results of clinical trials, AZD-1222 and Sputnik V vaccines have significant immunogenicity and safety, which produce antibodies against spike antigen [2, 16].

Sinopharm and Covaxin are inactivated virus particle vaccines, which are one of the oldest antiviral vaccine platforms. This method was developed in 1940 for influenza vaccine production [17]. This method is suitable for protection against some viruses [18]. The coronavirus particles are obtained from virus-infected cells and deactivated by means of chemical or physical techniques, including the use of UV, β-propiolactone, etc. [15, 17]. In this technique, it is important to choose the type of virus and an alum adjuvant is needed during injection [19–21]. Clinical trial outcomes confirm that these vaccines are safe and could stimulate impressive cellular and humoral immune responses [22, 23].

During the immediate development of a vaccine in a pandemic, it is critical that a protective response be established within a short period of time (e.g., < 1 month). In addition, previous research programs on vaccines (such as SARS-CoV14 and MERS-CoV13) revealed that both cellular and humoral immune responses are essential for an effective immune response [24].

Covid-19 infection usually stimulates neutralizing antibody production, and the rate of this response in people with COVID-19 infection is 50% and 100% on days 7 and 14 after the onset of symptoms, respectively [25]. On the other hand, serological tests are needed to evaluate the amount of neutralizing antibodies produced in a patient and also to recognize donors with high-neutralizing titers for convalescent plasma (CP) therapy [26]. For serum diagnosis, a number of COVID-19 analysis platforms have received FDA emergency usage permission, which determines the number of antibodies that bind to SARSCoV-2 spike protein. These methods include ELISA, lateral flow immunoassay, and microsphere immunoassay [26]. In addition, an ideal test should measure levels of neutralizing antibodies, which protect against re-infection, because not all spike-binding antibodies can inhibit viral infection [27].

It is critical to study vaccine efficiency during the general vaccination phase. In fact, genetic diversity in different human populations may affect the effectiveness of vaccines. The aim of this investigation was to evaluate the effectiveness of four available COVID-19 vaccines, including AZD1222 (AstraZeneca company), Sputnik V (Gamaleya Research Institute), BBIBP-CorV (Sinopharm), and Covaxin (Bharat Biotech company) in inducing anti-RBD Immunoglobulin G in a group of participants who received both doses of vaccine.

## Results

### Demographic characteristics

Overall, 214 participants (mean age: 36.5 ± 8.75, age range: 19–64 years, F/M ratio: 1.6) were registered in this project. The total number of health care employees in the Birjand hospitals was about 2500, and the vast majority of them had received two doses of the COVID-19 vaccine at the time of this study. About 10% of the vaccinated health care employees were included in this study. According to the Cochrane formula, the sample size was 330 people with a 95% confidence level. However, the number of available people who agreed to participate in this study was 280. Some of them did not participate in the second stage of blood sampling and some did not have the second dose of the vaccine. Finally, after screening, 214 people participated in the project. Details of demographic information and the frequency of vaccines are summarized in Table 1.

**Table 1:** Demographic characteristics of the recipients of vaccines

Variable	Outcome		AZD-1222	Sputnik V	Sinopharm	Covaxin	p Value
Type of vaccine			71 (33%)	57 (27%)	63 (29%)	23 (11%)	
Gender	Female (60%) Male (40%)		34 (48%) 37 (52%)	36 (63%) 21 (27%)	44 (70%) 19 (30%)	14 (61%) 9 (39%)	0.04
Age	< 50 years (86%) ≥ 50 years (14%)		61 (86%) 10 (14%)	46 (81%) 11 (19%)	54 (86%) 9 (14%)	23 (100%) 0 (0%)	0.01
Past Covid infection	14%		12%	7%	20%	17%	
	Female	15%					
	Male	12%					
	< 50 years	13%					
	≥ 50 years	18%					

^*^ One way ANOVA were used with a significance level of < 0.05

### COVID-19 vaccine efficiency

#### Comparison of the effectiveness of Sputnik V, AZD-1222, Sinopharm and Covaxin vaccines in all participants

**Fig. 1:**
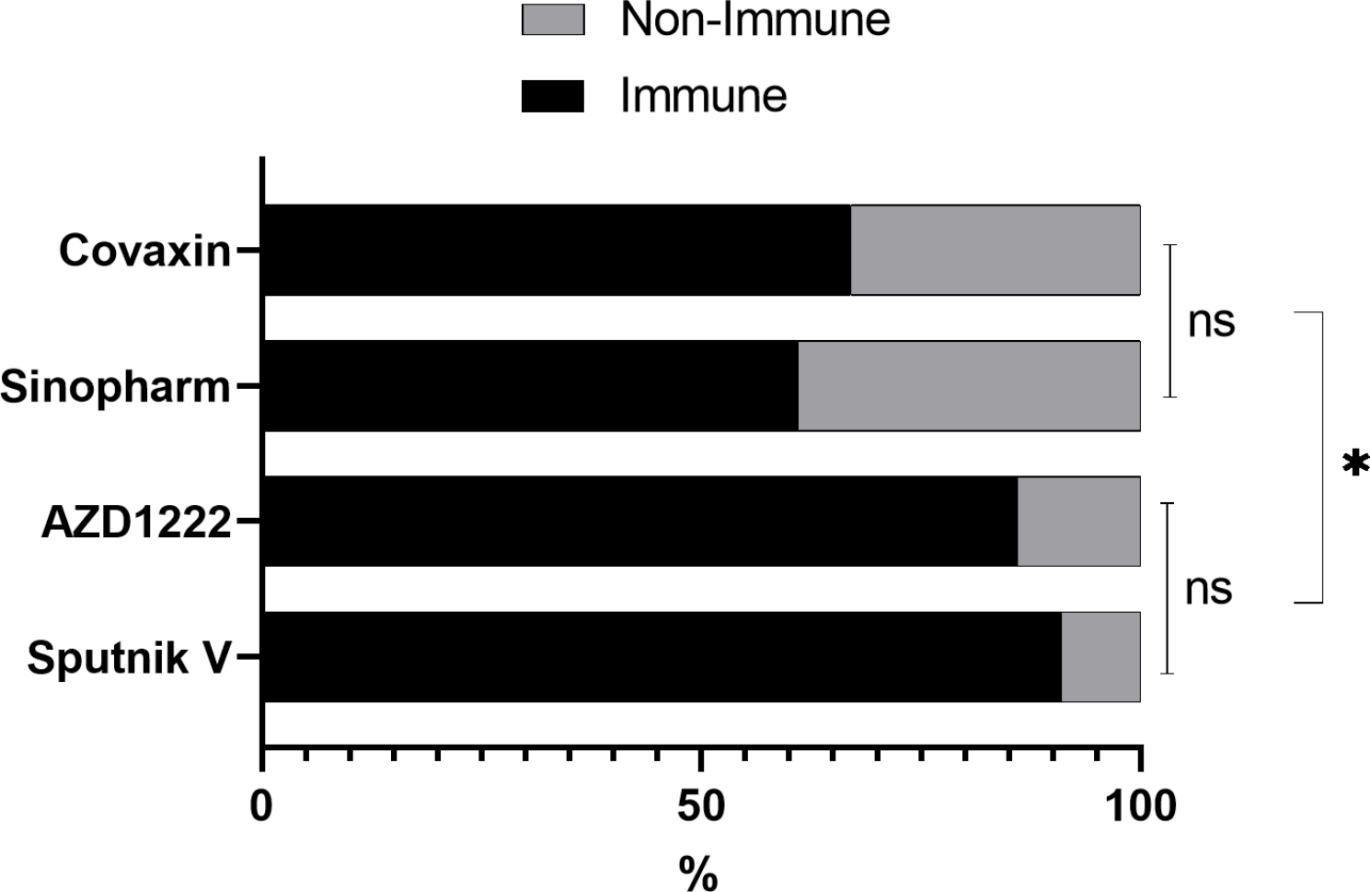
The frequency of immunized and unimmunized participants after receiving each of the vaccines

The frequency of IgG seropositivity for RBD protein two weeks after the second dose of vaccines was presented in Fig. 1; Table 2. Vector-based vaccines showed significantly higher efficacy than inactivated vaccines. A comparison of vaccine efficacy showed that there was no significant difference in immunogenicity between Sputnik V and AZD-1222. However, the production of neutralizing antibodies in Sinopharm and Covaxin vaccines is significantly lower than that of Sputnik V and AZD-1222 vaccines. Moreover, the immunogenicity of Sinopharm and Covaxin vaccines was not significantly different. In this comparison, people with a history of previous COVID-19 infection were excluded in order to eliminate any bias caused by previous infection.

**Table 2:** Comparison of immunogenicity in the four vaccines in the participants

Vaccines	Frequency of immunization	p Value			
		Sputnik V	AZD-1222	Sinopharm	Covaxin
Sputnik V	91%	-	0.69	0.01 ^*^	0.01 ^*^
AZD-1222	86%		-	0.02 ^*^	0.02 ^*^
Sinopharm	61%			-	0.73
Covaxin	67%				-

#### Relationship between vaccine efficacy vs. gender

**Fig. 2:**
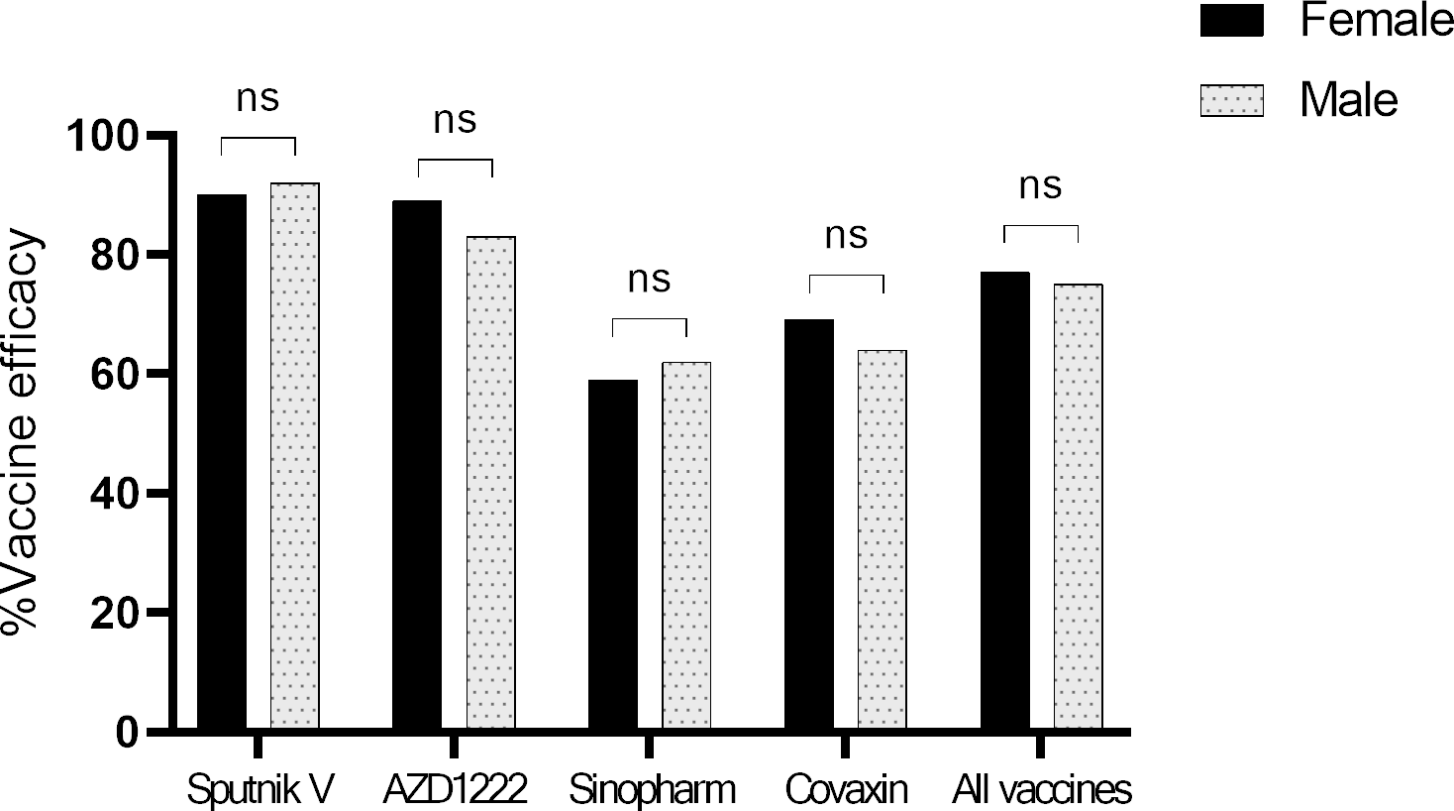
Comparison of immunogenicity in two groups of female and male groups with different vaccines

In general, there was no significant differences between males and females in rate of seropositivity (75% vs. 77%) although the rate was varied for different vaccines (Fig. 2).

#### Relationship between previous COVID19 infection and the efficiency of vaccines

**Fig. 3:**
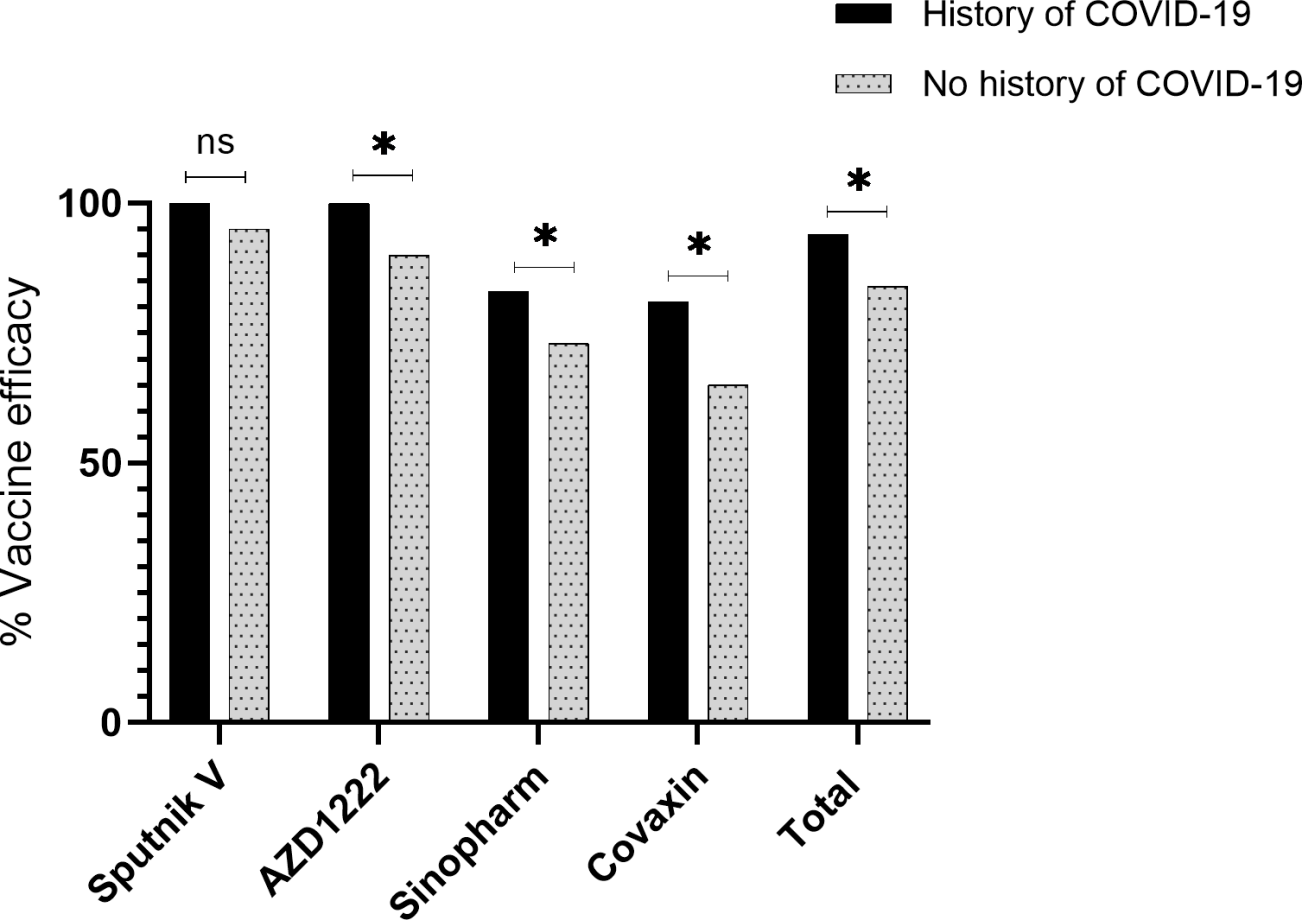
Relationship between previous COVID19 infection and the efficiency of vaccines. *p < 0.05, ns – not significant

The efficiency of the vaccine was evaluated based on a history of previous COVID-19 infection. The rate of seropositivity was significantly higher among convalescent patients in the case of AZD-1222, Sinopharm, and Covaxin vaccines, but the difference was not significant in the case of Sputnik V (Fig. 3).

## Discussion

Currently, vaccines are the best strategy for protection against COVID-19 infection. After the COVID-19 outbreak, several types of vaccines with different formulations were introduced and received by billions of people around the world. Induction of protective immunity by vaccines depends not only on host factors but also on vaccine components and structure, so it is necessary to evaluate the efficacy of different vaccines among people with different socio-economical and genetic backgrounds. In the current study, the rate of IgG seropositivity after receiving two doses of four different vaccines was evaluated in a group of participants. The Sputnik V vaccine, which was invented by Gamaleya Research Institute, has the gene for the SARS-CoV-2 glycoprotein S. The 1st and 2nd doses of the Sputnik V vaccine use two different types of adenoviruses as carriers of the spike gene; rAd26 and rAD5 for the first and second doses, respectively. Phase 1/2 clinical trials showed that both formulations of this vaccine were tolerable and safe [28]. “ChadOx1-nCoV-19” or AZD-1222, is composed of the replication-deficient simian adenovirus vector, which contains the sequence of the spike protein. According to studies, this vaccine is more tolerable in the elderly, and after a booster dose, it creates equal immunity in all age categories [28]. The BBIBP-CorV or Sinopharm vaccine is an inactivated whole virion, which induces high levels of neutralizing antibodies in six mammalian species and can protect them against SARS-CoV-2 infection [29]. BBV152, or Covaxin, is another inactivated whole SARS-CoV-2 virion particle, which is formulated with a toll-like receptor 7/8 agonist molecule adsorbed to alum. The Covaxin vaccine was developed using the NIV-2020-770 strain (obtained from an Indian patient with COVID-19), which has acceptable safety and can effectively elicit cellular and humoral responses [28].

According to the results, Sputnik V and AZD-1222 vaccines are more effective than Sinopharm and Covaxin vaccines. This may be due to differences in the platforms of these vaccines [21]. The platform used in Sputnik V and AZD-1222 vaccines is a live viral carrier, which according to previous studies has a high ability to stimulate the immune system [5, 24]. In contrast, the platform used in Sinopharm and Covaxin vaccines is inactive viruses that have less ability to stimulate the immune system [29, 30]. The immunogenicity of the Sputnik V and AZD-1222 vaccines was estimated to be about 91% and 86%, respectively, which is consistent with similar studies [31, 32]. Also, the effectiveness of Sinopharm and Covaxin vaccines was about 61% and 67%, respectively, which was consistent with most studies [30, 32]. The efficacy of the vaccine and the production of neutralizing antibodies in the Sputnik V and AZD-1222 vaccines were significantly higher than the Sinopharm and Covaxin vaccines. Given that the platform of Sputnik V and AZD-1222 vaccines is a type of live viral vector, it seems that the use of this platform has better immunogenic effects.

In a study by Voysey et al., AZD-1222 vaccine efficacy was determined in the UK, and vaccine efficacy was reported at 95.8%. Their result was slightly more than ours (86%) [33]. Ewer et al. determined the antibody responses induced by the AZD1222 vaccine in adults (mean age: 18–55) up to 8 weeks after vaccination. Robust immunity is induced against the spike antigen, as determined by total IgG ELISA. At day 14, the Anti-SARS-CoV-2 antibody was measurable and peaked at day 28 [34]. In another study, Wall and colleagues assessed AZD1222-induced neutralizing antibodies against the SARS-CoV-2 Delta variant of concern. Two doses of AZD1222 produced neutralizing antibodies against the wild type strain in all participants (100%). Moreover, 95% and 87% individuals had a measurable neutralizing antibody against the B.1.1.7 and D614G variants, respectively [35]. Jeewandara et al., measured immune responses to a single dose of the AZD1222 vaccine in healthcare workers. 93.4% of participants were positive for neutralizing antibody production, regardless of gender and age. Hemagglutination tests for antibodies to the RBD were done in a sub-cohort, and ACE2 blocking antibodies were detected in 97.1% of naive people [36]. Moreover, Wall et al. investigated the ability of AZD1222 vaccination to elicit neutralizing antibodies against SARS-CoV-2 (Delta) in 106 participants. According to the result, 87% of individuals had measurable neutralizing antibodies against the B.1.1.7 and D614G variants, but only 62% of participants had quantifiable NAbTs against B.1.617.2 (Delta variant) following two doses of AZD1222 [37].

In a study by Logunov et al., Sputnik V vaccine efficacy was determined among adult participants. Vaccine efficacy in this study was 92%, which was very similar to our study (91%) [24]. Moreover, in a study by Claro et al., they assessed the antibody (IgG) response against the RBD of the spike protein and the Nucleocapsid protein (NP) in Venezuela after the vaccination by Sputnik V. Antibody responses against RBD and nucleocapsid protein were measured by ELISA. All of the participants demonstrated a strong IgG immune response against RBD after the second dose, but only 58% of participants had an immune response after the first dose [38]. In another study by Rossi and colleagues, among health care workers in Argentina, SARS-CoV-2 specific antibody responses were evaluated after vaccination by Sputnik V. IgG anti-spike titers and neutralizing capacity were determined after two doses, and 94% of participants developed spike-specific IgG antibodies. Interestingly, a single Sputnik V dose elicited higher antibody levels in previously infected individuals [39]. Also, Gushchin et al. evaluated the neutralizing activity of sera from Sputnik V vaccinated subjects against variants of concern, such as the alpha variant. The data obtained indicated no significant differences in virus-neutralizing activity against the alpha variant [40].

There are many studies on the effect of the Sinopharm vaccine on the creation of neutralizing antibodies against SARS-CoV-2. Holt et al., performed a study to evaluate the antibody responses following vaccination with the Sinopharm vaccine in the UAE after two doses (1296 participants). The antibody responses were measured 14–21 days after the second dose by means of chemiluminescence immunoassay technology, and neutralizing antibody testing was carried out by a blocking enzyme-linked immunosorbent assay. According to the result, 56% of participants had a positive anti-spike antibody against SARS-CoV-2, which was almost similar to ours (61%) [41]. In another study by Jeewandara et al., the kinetics of immune responses following the Sinopharm/BBIBP-CorV was measured in Sri Lankans. SARS-CoV-2 specific total antibodies were evaluated in 83 individuals by ELISA, after the second dose. In their study, RBD specific antibodies were measured by ELISA, and about 95% of participants had measurable SARS-CoV-2 specific total antibodies [42]. Moreover, Ferenc and colleagues determined virus neutralizing antibody responses after the second dose of Sinopharm Covid-19 vaccine in 450 participants. Outcomes were examined in a multivariable model for gender and age. In a similar vein to our study, gender was slightly correlated with the antibody titers [43]. Similar to our study, gender had no significant effect on the efficacy of Sinopharm vaccine.

Various studies have been performed on the efficacy of the Covaxin vaccine. In a study by Ella, Covaxin vaccine efficacy was measured in Indian hospitals. Participants were followed two weeks after the second vaccination, and vaccine efficacy was reported at 77.8%, which was slightly more than our results (67%) [30]. In another study by Singh et al., antibody response was determined after the Covaxin (BBV-152) vaccine among 515 healthcare employees in India. An anti-spike antibody titer was measured on day 21 after vaccination. The IgG to SARS-CoV-2 directed against the spike protein was assayed with an indirect chemiluminescence immunoassay (CLIA). About 44% of participants showed seropositivity after vaccination, which was almost similar to our result (70%). Also similar to our study, no difference was observed with gender [44]. In a study by Kumar, antibody responses to the BBV152 vaccine were measured in healthcare professionals. Serological testing for anti-spike antibody measurement was performed using a chemiluminescence immunoassay. According to their results, about 76% of participants showed seropositivity after vaccination, which is higher than our result (67%) [45].

Finally, in another study, Covid-19 vaccine efficacy was done by Siddique and Ahmed in Pakistan. In this study, the efficacy of various vaccines, including Sputnik V, AZD-1222, and Sinopharm vaccines, was evaluated. The results showed that the efficacy of Sputnik V, AZD-1222, and Sinopharm vaccines was 92%, 70%, and 79%, respectively, which compared to our study. However, the efficacy of AZD-1222 and Sinopharm vaccine in this study was reported to be lower and higher than our study, respectively [46].

## Conclusion

The results of this study showed that vector-based vaccines have more efficacy in producing humoral immunity than inactivated vaccines, and gender does not affect the effectiveness of these vaccines. Further studies need to evaluate the duration of protection immunity acquired by COVID-19 vaccines.

## Methods

### Design study

From May to August 2021, personnel of Birjand University of Medical Sciences, including healthcare workers, students, and administrative staff, who wanted to receive the COVID-19 vaccine were invited to participate in the study. Participants donated 5 milliliters of their venous blood before receiving the first dose of vaccine and two weeks after the second dose and completed an online questionnaire. The questionnaire consisted of questions about demographic data, the history of previous COVID-19 infection, as well as the date and type of received vaccines. Sera from collected blood were separated by centrifugation and stored at -20^o^C until analysis. To evaluate the vaccine-induced humoral response against COVID-19, sera were checked for anti-RBD neutralizing antibody by PT-SARS-CoV-2-Neutralizing-Ab-96 (Pishtazteb Co., Tehran, Iran) kit, in duplicate.

X neutralizing antibody.

### Calculate the immunological status ratio (ISR) of the sample

The amount of ISR was obtained by dividing the optical density (OD) of each sample by the cut-off value. ISR was calculated according to the following formula. Considering that the experiments were performed in duplicate, to calculate the ISR, the average OD of the samples was first calculated and then divided by the cut-off value. According to the kit instructions, ISR values greater than 1.1, less than 0.8, and those values in between were considered positive, negative, and borderline, respectively. An ISR between 0.8 and 1.1 was considered borderline according to the kit instructions, and these cases were repeated. In the new experiments, results ≥ 1 and results < 1 were considered as immunized and non-immunized, respectively.

$$IRS = \frac{{OD\,of\,the\,clinical\,sample}}{{Cut\,off\, = \,0.22 + OD\,of\,Negative\,control}}$$

### Inclusion/exclusion criteria

The inclusion criteria for this study were injections of both doses of one of the vaccines, Sputnik V, AZD-1222, Covaxin, and Sinopharm. The exclusion criteria were having a positive COVID-19 test through the study period and being unwilling to donate blood on time.

### Ethical approval

This study was accepted on April 17, 2021 by the Ethics Committee of the Birjand University of Medical University (IR.BUMS.REC.1400.027), and all participants filled out the consent form.

### Statistical analysis

The data was analyzed by the SPSS software version 22.0 (SPSS Inc., Chicago, IL, USA). The Chi-square test and Student’s t-test with a significance value p < 0.05 were used.

## Electronic supplementary material

Below is the link to the electronic supplementary material.

Supplementary Material 1

## Data Availability

Not applicable.

## References

[1] Zhu F-C, Li Y-H, Guan X-H, Hou L-H, Wang W-J, Li J-X, Wu S-P, Wang B-S, Wang Z, Wang L (2020). Safety, tolerability, and immunogenicity of a recombinant adenovirus type-5 vectored COVID-19 vaccine: a dose-escalation, open-label, non-randomised, first-in-human trial. The Lancet.

[2] Zhu F-C, Guan X-H, Li Y-H, Huang J-Y, Jiang T, Hou L-H, Li J-X, Yang B-F, Wang L, Wang W-J (2020). Immunogenicity and safety of a recombinant adenovirus type-5-vectored COVID-19 vaccine in healthy adults aged 18 years or older: a randomised, double-blind, placebo-controlled, phase 2 trial. The Lancet.

[3] Matta S, Chopra KK, Arora VK. Morbidity and mortality trends of Covid 19 in top 10 countries, indian journal of tuberculosis (2020). 10.1016/j.ijtb.2020.09.031 PMC7543896 33308665

[4] Zhu N, Zhang D, Wang W, Li X, Yang B, Song J, Zhao X, Huang B, Shi W, Lu R, A novel coronavirus from patients with pneumonia in China, 2019, New England journal of medicine (2020). 10.1056/NEJMoa2001017 PMC7092803 31978945

[5] Folegatti PM, Ewer KJ, Aley PK, Angus B, Becker S, Belij-Rammerstorfer S, Bellamy D, Bibi S, Bittaye M, Clutterbuck EA (2020). Safety and immunogenicity of the ChAdOx1 nCoV-19 vaccine against SARS-CoV-2: a preliminary report of a phase 1/2, single-blind, randomised controlled trial. The Lancet.

[6] Raveendran A (2021). Long COVID-19: Challenges in the diagnosis and proposed diagnostic criteria. Diabetes & Metabolic Syndrome.

[7] Salamanna F, Veronesi F, Martini L, Landini MP, Fini M (2021). Post-COVID-19 Syndrome: The Persistent Symptoms at the Post-viral Stage of the Disease. A Systematic Review of the Current Data. Front Med.

[8] Parkin A, Davison J, Tarrant R, Ross D, Halpin S, Simms A, Salman R, Sivan M (2021). A Multidisciplinary NHS COVID-19 Service to Manage Post-COVID-19 Syndrome in the Community. J Prim Care Community Health.

[9] Walsh EE, Frenck RW, Falsey AR, Kitchin N, Absalon J, Gurtman A, Lockhart S, Neuzil K, Mulligan MJ, Bailey R (2020). Safety and immunogenicity of two RNA-based Covid-19 vaccine candidates. N Engl J Med.

[10] Amanat F, Krammer F (2020). SARS-CoV-2 vaccines: status report. Immunity.

[11] Hossain MJ, Kuddus MR, Rashid MA, Sultan MZ (2021). Understanding and dealing the SARS-COV-2 infection: an updated concise review. Bangladesh Pharm J.

[12] Zare H, Rezapour H, Mahmoodzadeh S, Fereidouni M (2021). Prevalence of COVID-19 vaccines (Sputnik V, AZD-1222, and Covaxin) side effects among healthcare workers in Birjand city. Iran Int Immunopharmacol.

[13] Mackett M, Smith GL, Moss B, Vaccinia virus: a selectable eukaryotic cloning and expression vector, Proceedings of the National Academy of Sciences 79(23) (1982) 7415–7419. 10.1073/pnas.79.23.7415 PMC347350 6296831

[14] Jackson DA, Symons RH, Berg P, Biochemical method for inserting new genetic information into DNA of Simian Virus 40: circular SV40 DNA molecules containing lambda phage genes and the galactose operon of Escherichia coli, Proceedings of the National Academy of Sciences 69(10) (1972) 2904–2909. 10.1073/pnas.69.10.2904 PMC389671 4342968

[15] Fathizadeh H, Afshar S, Masoudi MR, Gholizadeh P, Asgharzadeh M, Ganbarov K, Köse Ş, Yousefi M, Kafil HS. SARS-CoV-2 (Covid-19) vaccines structure, mechanisms and effectiveness: A review, International Journal of Biological Macromolecules (2021). 10.1016/j.ijbiomac.2021.08.076 PMC8364403 34403674

[16] Xia S, Duan K, Zhang Y, Zhao D, Zhang H, Xie Z, Li X, Peng C, Zhang Y, Zhang W (2020). Effect of an inactivated vaccine against SARS-CoV-2 on safety and immunogenicity outcomes: interim analysis of 2 randomized clinical trials. JAMA.

[17] BARBERIS I, MYLES P, Ault S, Bragazzi N, Martini M (2016). History and evolution of influenza control through vaccination: from the first monovalent vaccine to universal vaccines. J Prev Med Hyg.

[18] Liu C, Mendonça L, Yang Y, Gao Y, Shen C, Liu J, Ni T, Ju B, Liu C, Tang X (2020). The architecture of inactivated SARS-CoV-2 with postfusion spikes revealed by cryo-EM and cryo-ET. Structure.

[19] Kumar A, Meldgaard TS, Bertholet S (2018). Novel platforms for the development of a universal influenza vaccine. Front Immunol.

[20] Gao Q, Bao L, Mao H, Wang L, Xu K, Yang M, Li Y, Zhu L, Wang N, Lv Z (2020). Development of an inactivated vaccine candidate for SARS-CoV-2. Science.

[21] Singh AK, Phatak SR, Singh R, Bhattacharjee K, Singh NK, Gupta A, Sharma A. Antibody Response after Second-dose of ChAdOx1-nCOV (CovishieldTM) and BBV-152 (CovaxinTM) among Health Care Workers in India: Final Results of Cross-sectional Coronavirus Vaccine-induced Antibody Titre (COVAT) study, medRxiv (2021). 10.1016/j.vaccine.2021.09.055 PMC8461292 34600747

[22] Sapkal GN, Yadav P, Ella R, Deshpande G, Sahay R, Gupta N, Mohan VK, Abraham P, Panda S, Bhargava B, Neutralization of UK-variant VUI-202012/01 with COVAXIN vaccinated human serum, BioRxiv (2021).

[23] Kim JH, Marks F, Clemens JD (2021). Looking beyond COVID-19 vaccine phase 3 trials. Nat Med.

[24] Logunov DY, Dolzhikova IV, Zubkova OV, Tukhvatullin AI, Shcheblyakov DV, Dzharullaeva AS, Grousova DM, Erokhova AS, Kovyrshina AV, Botikov AG (2020). Safety and immunogenicity of an rAd26 and rAd5 vector-based heterologous prime-boost COVID-19 vaccine in two formulations: two open, non-randomised phase 1/2 studies from Russia. The Lancet.

[25] Huang AT, Garcia-Carreras B, Hitchings MD, Yang B, Katzelnick LC, Rattigan SM, Borgert BA, Moreno CA, Solomon BD, Rodriguez-Barraquer I, A systematic review of antibody mediated immunity to coronaviruses: antibody kinetics, correlates of protection, and association of antibody responses with severity of disease, MedRxiv (2020). 10.1038/s41467-020-18450-4 PMC7499300 32943637

[26] Muruato AE, Fontes-Garfias CR, Ren P, Garcia-Blanco MA, Menachery VD, Xie X, Shi P-Y (2020). A high-throughput neutralizing antibody assay for COVID-19 diagnosis and vaccine evaluation. Nat Commun.

[27] Cao X, Li W, Wang T, Ran D, Davalos V, Planas-Serra L, Pujol A, Esteller M, Wang X, Yu H. Accelerated biological aging in COVID-19 patients, Nature communications (2022). 10.1038/s41467-022-29801-8 PMC9018863 35440567

[28] Yan Z-P, Yang M, Lai C-L (2021). COVID-19 Vaccines: A Review of the Safety and Efficacy of Current Clinical Trials. Pharmaceuticals.

[29] Wang H, Zhang Y, Huang B, Deng W, Quan Y, Wang W, Xu W, Zhao Y, Li N, Zhang J (2020). Development of an inactivated vaccine candidate, BBIBP-CorV, with potent protection against SARS-CoV-2. Cell.

[30] Ella R, Reddy S, Jogdand H, Sarangi V, Ganneru B, Prasad S, Das D, Raju D, Praturi U, Sapkal G. Safety and immunogenicity of an inactivated SARS-CoV-2 vaccine, BBV152: interim results from a double-blind, randomised, multicentre, phase 2 trial, and 3-month follow-up of a double-blind, randomised phase 1 trial. The Lancet Infectious Diseases; 2021. 10.1016/S1473-3099(21)00070-0 PMC8221739 33705727

[31] Ghiasi N, Valizadeh R, Arabsorkhi M, Hoseyni TS, Esfandiari K, Sadighpour T, Jahantigh HR, Efficacy and side effects of Sputnik V, Sinopharm and AstraZeneca vaccines to stop COVID-19; a review and discussion, (2021).

[32] Siddique S, Ahmed S (2021). COVID-19 Vaccines in Pakistan: Efficacy, Adverse Effects and Availability. J Islamabad Med Dent Coll.

[33] Voysey M, Clemens SAC, Madhi SA, Weckx LY, Folegatti PM, Aley PK, Angus B, Baillie VL, Barnabas SL, Bhorat QE (2021). Safety and efficacy of the ChAdOx1 nCoV-19 vaccine (AZD1222) against SARS-CoV-2: an interim analysis of four randomised controlled trials in Brazil, South Africa, and the UK. The Lancet.

[34] Ewer KJ, Barrett JR, Belij-Rammerstorfer S, Sharpe H, Makinson R, Morter R, Flaxman A, Wright D, Bellamy D, Bittaye M (2021). T cell and antibody responses induced by a single dose of ChAdOx1 nCoV-19 (AZD1222) vaccine in a phase 1/2 clinical trial. Nat Med.

[35] Wall EC, Wu M, Harvey R, Kelly G, Warchal S, Sawyer C, Daniels R, Adams L, Hobson P, Hatipoglu E (2021). AZD1222-induced neutralising antibody activity against SARS-CoV-2 Delta VOC. The Lancet.

[36] Jeewandara C, Kamaladasa A, Pushpakumara PD, Jayathilaka D, Aberathna IS, Danasekara DRSR, Guruge D, Ranasinghe T, Dayarathna S, Pathmanathan T (2021). Immune responses to a single dose of the AZD1222/Covishield vaccine in health care workers. Nat Commun.

[37] Wall EC, Wu M, Harvey R, Kelly G, Warchal S, Sawyer C, Daniels R, Adams L, Hobson P, Hatipoglu E (2021). Ability of AZD1222 vaccination to elicit neutralising antibodies against SARS-CoV-2 VOC B. 1.617. 2 (Delta). Lancet (London England).

[38] Claro F, Silva D, Rodriguez M, Rangel HR, de Waard JH (2021). Immunoglobulin G antibody response to the Sputnik V vaccine: previous SARS-CoV-2 seropositive individuals may need just one vaccine dose. Int J Infect Dis.

[39] Rossi AH, Ojeda DS, Varese A, Sanchez L, Ledesma MMGL, Mazzitelli I, Juliá AA, Rouco SO, Pallarés HM, Navarro GSC (2021). Sputnik V vaccine elicits seroconversion and neutralizing capacity to SARS-CoV-2 after a single dose. Cell Rep Med.

[40] Gushchin VA, Dolzhikova IV, Shchetinin AM, Odintsova AS, Siniavin AE, Nikiforova MA, Pochtovyi AA, Shidlovskaya EV, Kuznetsova NA, Burgasova OA (2021). Neutralizing activity of sera from Sputnik V-vaccinated people against variants of concern (VOC: B. 1.1. 7, B. 1.351, P. 1, B. 1.617. 2, B. 1.617. 3) and Moscow endemic SARS-CoV-2 variants. Vaccines.

[41] Holt SG, Mahmoud S, Ahmed W, Acuna JM, Al Madani AK, Eltantawy I, Zaher WA, Goodier GJ, Al Kaabi NA, Al AA, Obaidli, An analysis of antibody responses and clinical sequalae of the Sinopharm HB02 COVID19 vaccine in dialysis patients in the United Arab Emirates, Nephrology (2021). 10.1111/nep.13980 PMC8646272 34569677

[42] Jeewandara C, Aberathna I, Pushpakumara P, Kamaladasa A, Guruge D, Wijesinghe A, Gunasekara B, Tanussiya S, Kuruppu H, Ranasinghe T, Persistence of antibody and T cell responses to the Sinopharm/BBIBP-CorV vaccine in Sri Lankan individuals, Medrxiv (2021).

[43] Ferenci T, Sarkadi B, Virus neutralizing antibody responses after two doses of BBIBP-CorV (Sinopharm, Beijing CNBG) vaccine, medRxiv (2021). 10.1186/s12879-022-07069-z PMC8785690 35073866

[44] Singh AK, Phatak SR, Singh R, Bhattacharjee K, Singh NK, Gupta A, Sharma A (2021). Antibody response after first and second-dose of ChAdOx1-nCOV (CovishieldTM®) and BBV-152 (CovaxinTM®) among health care workers in India: The final results of cross-sectional coronavirus vaccine-induced antibody titre (COVAT) study. Vaccine.

[45] Kumar NP, Padmapriyadarsini C, Devi KU, Banurekha V, Nancy A, Kumar CG, Murhekar MV, Gupta N, Panda S, Babu S (2021). Antibody responses to the BBV152 vaccine in individuals previously infected with SARS-CoV-2: A pilot study. Indian J Med Res.

[46] Siddique S, Ahmed S, COVID-19 Vaccines in Pakistan: Efficacy, Adverse Effects and Availability, JOURNAL OF ISLAMABAD MEDICAL & DENTAL COLLEGE 10(2) (2021) 125–130.

