# Supplementary material for: Analysis and comparison of anti-RBD neutralizing antibodies from AZD-1222, Sputnik V, Sinopharm and Covaxin vaccines and its relationship with gender among health care workers
Source: Immun Ageing. 2022 Oct 22;19:47. doi: 10.1186/s12979-022-00303-x (PMC9587595; doi:10.1186/s12979-022-00303-x)
Supplement: Supplementary file 1 — Supplementary Material 1 [file 12979_2022_303_MOESM1_ESM.docx]

Analysis and comparison of anti-RBD neutralizing antibodies from AZD-1222, Sputnik V, Sinopharm and Covaxin vaccines and its relationship with gender among health care workers

Hamed Zare ^a^, Hadis Rezapour ^b^, Alireza Fereidouni ^c^, Saboura Nikpour ^b^, Sara Mahmoudzadeh ^b^, Simon G Royce ^d^, Mohammad Fereidouni ^c, b*^

a, Pharmaceutical Sciences and Cosmetic Products Research Center, Kerman University of Medical Sciences, Kerman, Iran

b, Department of Immunology, Faculty of Medicine, Birjand University of Medical Sciences, Birjand, Iran

c, Cellular and Molecular Research Center, Birjand University of Medical Sciences, Birjand, Iran

d, Department of Pharmacology, Monash University, Clayton, Australia

*** Corresponding author**: Mohammad Fereidouni

Email address: [Dr.m.fereidouni@gmail.com](mailto:Dr.m.fereidouni@gmail.com)

Postal address: Cellular and Molecular Research Center, Birjand University of Medical Sciences, Birjand, Iran.

Phone number: +985632433004

**Abstract**

**Background:** Vaccine efficiency has a significant role in the public perception of vaccination. The current study was designed to evaluate the efficacy of COVID-19 vaccines (AZD-1222, Sputnik-V, Sinopharm, and Covaxin) and the effect of gender on vaccine efficacy. We evaluated the efficacy of these vaccines among 214 health care employees in Iran. Blood samples were taken from all participants on day 0 and 14 days after the second dose. Humoral responses were evaluated by the PT-SARS-CoV-2-Neutralizing-Ab-96.

**Results:** The frequency of immunized individuals in the Sputnik V and AZD-1222 groups was 91% and 86%, respectively. This rate was 61% and 67% for Sinopharm and Covaxin vaccines. A comparison of the results obtained from the effectiveness of the vaccines between female and male groups did not demonstrate a significant difference.

**Conclusions:** According to the results, Sputnik V and AZD-1222 vaccines were more effective than Sinopharm and Covaxin vaccines. Moreover, the effectiveness of these vaccines is not related to gender

**Key words:** Vaccine, COVID-19, Sputnik V, AZD-1222, Sinopharm, Covaxin.

***1. Introduction***

SARS-CoV-2 was first recognized as a COVID-19 agent two years ago (December 2019) [1-3]. SARS-CoV-2 has infected more than 595 million individuals worldwide (Aug 2022) and has been responsible for more than 6.45 million deaths [4]. SARS-CoV-2 infection usually causes a wide range of symptoms in people, which can range from mild symptoms to severe manifestations and even death. Also, people who have survived severe infections suffer from post-COVID-19 syndrome such as fatigue, shortness of breath, muscle pains, etc. [5-7]. In addition, convalescent patients may develop other infections (bacterial, viral, and fungal), cardiovascular problems, and other psychological issues [6-8]. Due to the health and economic pressures of the COVID-19 epidemic, vaccination is able to reduce this burden by decreasing the mortality rate of SARS-CoV-2 infection [9, 10].

According to the World Health Organization, more than 137 candidates are currently undergoing clinical development, of which a small number are licensed and approved [11]. The characteristics of an ideal vaccine are: effectiveness after one or two doses of vaccination; protection of target populations such as the elderly and people with underlying disease; efficacy and protection for at least 6 months; and reduce further transmission of the virus to others [5, 12].

AZD-1222 (Oxford/AstraZeneca), BBIBP-CorV (Sinopharm), Sputnik V (Gamaleya Research Institute) and Covaxin (Bharat Biotech) vaccines were investigated in the current study (February to March 2021).

AZD-1222 and Sputnik V are based on a non-replicating adenoviral vector platform. This platform is based on adenoviruses, which are deactivated by removing the E1B and E1A genes, and was established in 1972 [13, 14]. The spike antigen cDNA is inserted into a non-replicating adenoviral vector, and then these vaccines provide the cDNA of the spike protein to infected cells, which leads to spike protein expression in host cells [15]. AZD-1222 and Sputnik V elevate both humoral and cellular immunity [15]. According to the results of clinical trials, AZD-1222 and Sputnik V vaccines have significant immunogenicity and safety, which produce antibodies against spike antigen [2, 16].

Sinopharm and Covaxin are inactivated virus particle vaccines, which are one of the oldest antiviral vaccine platforms. This method was developed in 1940 for influenza vaccine production [17]. This method is suitable for protection against some viruses [18]. The coronavirus particles are obtained from virus-infected cells and deactivated by means of chemical or physical techniques, including the use of UV, β-propiolactone, etc. [15, 17]. In this technique, it is important to choose the type of virus and an alum adjuvant is needed during injection [19, 20, 21]. Clinical trial outcomes confirm that these vaccines are safe and could stimulate impressive cellular and humoral immune responses [22, 23].

During the immediate development of a vaccine in a pandemic, it is critical that a protective response be established within a short period of time (e.g., < 1 month). In addition, previous research programs on vaccines (such as SARS-CoV14 and MERS-CoV13) revealed that both cellular and humoral immune responses are essential for an effective immune response [24].

Covid-19 infection usually stimulates neutralizing antibody production, and the rate of this response in people with COVID-19 infection is 50% and 100% on days 7 and 14 after the onset of symptoms, respectively [25]. On the other hand, serological tests are needed to evaluate the amount of neutralizing antibodies produced in a patient and also to recognize donors with high-neutralizing titers for convalescent plasma (CP) therapy [26]. For serum diagnosis, a number of COVID-19 analysis platforms have received FDA emergency usage permission, which determines the number of antibodies that bind to SARSCoV-2 spike protein. These methods include ELISA, lateral flow immunoassay, and microsphere immunoassay [26]. In addition, an ideal test should measure levels of neutralizing antibodies, which protect against re-infection, because not all spike-binding antibodies can inhibit viral infection [27].

It is critical to study vaccine efficiency during the general vaccination phase. In fact, genetic diversity in different human populations may affect the effectiveness of vaccines. The aim of this investigation was to evaluate the effectiveness of four available COVID-19 vaccines, including AZD1222 (AstraZeneca company), Sputnik V (Gamaleya Research Institute), BBIBP-CorV (Sinopharm), and Covaxin (Bharat Biotech company) in inducing anti-RBD Immunoglobulin G in a group of participants who received both doses of vaccine.

***2. Results***

***2.1. Demographic Characteristics***

Overall, 214 participants (mean age: 36.5 ± 8.75, age range: 19-64 years, F/M ratio: 1.6) were registered in this project. The total number of health care employees in the Birjand hospitals was about 2500, and the vast majority of them had received two doses of the COVID-19 vaccine at the time of this study. About 10% of the vaccinated health care employees were included in this study. According to the Cochrane formula, the sample size was 330 people with a 95% confidence level. However, the number of available people who agreed to participate in this study was 280. Some of them did not participate in the second stage of blood sampling and some did not have the second dose of the vaccine. Finally, after screening, 214 people participated in the project. Details of demographic information and the frequency of vaccines are summarized in Table 1.

Table 1: Demographic characteristics of the recipients of vaccines

| Variable | Outcome | | AZD-1222 | Sputnik V | Sinopharm | Covaxin | *pValue* |
| --- | --- | --- | --- | --- | --- | --- | --- |
| Type of vaccine |  | | 71 (33 %) | 57 (27 %) | 63 (29 %) | 23 (11 %) |  |
| Gender | Female (60 %)  Male (40 %) | | 34 (48 %)  37 (52 %) | 36 (63 %)  21 (27 %) | 44 (70 %)  19 (30 %) | 14 (61 %)  9 (39 %) | 0.04 |
| Age | < 50 years (86 %)  ≥ 50 years (14 %) | | 61 (86 %)  10 (14 %) | 46 (81 %)  11 (19 %) | 54 (86 %)  9 (14 %) | 23 (100%)  0 (0 %) | 0.01 |
| Past Covid infection | 14 % | | 12 % | 7 % | 20 % | 17 % |  |
|  | Female | 15 % |  | | | | |
|  | Male | 12 % |  |  |  |  |  |
|  |  | | | | | | |
|  | < 50 years | 13 % |  | | | | |
|  | ≥ 50 years | 18 % |  |  |  |  |  |

^*^ One way ANOVA were used with a signiﬁcance level of <0.05.

***2.2. COVID-19 vaccine efficiency***

*2.2.1. Comparison of the effectiveness of Sputnik V, AZD-1222, Sinopharm and Covaxin vaccines in all participants*

The frequency of IgG seropositivity for RBD protein two weeks after the second dose of vaccines was presented in Figure 1 and Table 2. Vector-based vaccines showed significantly higher efficacy than inactivated vaccines. A comparison of vaccine efficacy showed that there was no significant difference in immunogenicity between Sputnik V and AZD-1222. However, the production of neutralizing antibodies in Sinopharm and Covaxin vaccines is significantly lower than that of Sputnik V and AZD-1222 vaccines. Moreover, the immunogenicity of Sinopharm and Covaxin vaccines was not significantly different. In this comparison, people with a history of previous COVID-19 infection were excluded in order to eliminate any bias caused by previous infection.

Figure 1: The frequency of immunized and unimmunized participants after receiving each of the vaccines.

Table 2: Comparison of immunogenicity in the four vaccines in the participants.

| pValue | | | | Frequency of immunization | Vaccines |
| --- | --- | --- | --- | --- | --- |
| Covaxin | Sinopharm | AZD-1222 | Sputnik V |  |  |
| 0.01 ^*^ | 0.01 ^*^ | 0.69 | - | 91 % | Sputnik V |
| 0.02 ^*^ | 0.02 ^*^ | - |  | 86 % | AZD-1222 |
| 0.73 | - |  |  | 61 % | Sinopharm |
| - |  |  |  | 67 % | Covaxin |

*2.2.2. Relationship between vaccine efficacy vs. gender*

In general, there was no significant differences between males and females in rate of seropositivity (75% vs. 77%) although the rate was varied for different vaccines (Figure 2).

Figure 2: Comparison of immunogenicity in two groups of female and male groups with different vaccines.

*2.2.3. Relationship between previous COVID19 infection and the efficiency of vaccines*

The efficiency of the vaccine was evaluated based on a history of previous COVID-19 infection. The rate of seropositivity was significantly higher among convalescent patients in the case of AZD-1222, Sinopharm, and Covaxin vaccines, but the difference was not significant in the case of Sputnik V (Figure 3).

Figure 3: Relationship between previous COVID19 infection and the efficiency of vaccines. *p<0.05, ns – not significant

**3. Discussion**

Currently, vaccines are the best strategy for protection against COVID-19 infection. After the COVID-19 outbreak, several types of vaccines with different formulations were introduced and received by billions of people around the world. Induction of protective immunity by vaccines depends not only on host factors but also on vaccine components and structure, so it is necessary to evaluate the efficacy of different vaccines among people with different socio-economical and genetic backgrounds. In the current study, the rate of IgG seropositivity after receiving two doses of four different vaccines was evaluated in a group of participants. The Sputnik V vaccine, which was invented by Gamaleya Research Institute, has the gene for the SARS-CoV-2 glycoprotein S. The 1st and 2nd doses of the Sputnik V vaccine use two different types of adenoviruses as carriers of the spike gene; rAd26 and rAD5 for the first and second doses, respectively. Phase 1/2 clinical trials showed that both formulations of this vaccine were tolerable and safe [28]. “ChadOx1-nCoV-19” or AZD-1222, is composed of the replication-deficient simian adenovirus vector, which contains the sequence of the spike protein. According to studies, this vaccine is more tolerable in the elderly, and after a booster dose, it creates equal immunity in all age categories [28]. The BBIBP-CorV or Sinopharm vaccine is an inactivated whole virion, which induces high levels of neutralizing antibodies in six mammalian species and can protect them against SARS-CoV-2 infection [29]. BBV152, or Covaxin, is another inactivated whole SARS-CoV-2 virion particle, which is formulated with a toll-like receptor 7/8 agonist molecule adsorbed to alum. The Covaxin vaccine was developed using the NIV-2020-770 strain (obtained from an Indian patient with COVID-19), which has acceptable safety and can effectively elicit cellular and humoral responses [28].

According to the results, Sputnik V and AZD-1222 vaccines are more effective than Sinopharm and Covaxin vaccines. This may be due to differences in the platforms of these vaccines [21]. The platform used in Sputnik V and AZD-1222 vaccines is a live viral carrier, which according to previous studies has a high ability to stimulate the immune system [5, 24]. In contrast, the platform used in Sinopharm and Covaxin vaccines is inactive viruses that have less ability to stimulate the immune system [29, 30]. The immunogenicity of the Sputnik V and AZD-1222 vaccines was estimated to be about 91% and 86%, respectively, which is consistent with similar studies [31, 32]. Also, the effectiveness of Sinopharm and Covaxin vaccines was about 61% and 67%, respectively, which was consistent with most studies [30, 32]. The efficacy of the vaccine and the production of neutralizing antibodies in the Sputnik V and AZD-1222 vaccines were significantly higher than the Sinopharm and Covaxin vaccines. Given that the platform of Sputnik V and AZD-1222 vaccines is a type of live viral vector, it seems that the use of this platform has better immunogenic effects.

In a study by Voysey *et al*., AZD-1222 vaccine efficacy was determined in the UK, and vaccine efficacy was reported at 95.8%. Their result was slightly more than ours (86%) [33]. Ewer *et al.* determined the antibody responses induced by the AZD1222 vaccine in adults (mean age: 18–55) up to 8 weeks after vaccination. Robust immunity is induced against the spike antigen, as determined by total IgG ELISA. At day 14, the Anti-SARS-CoV-2 antibody was measurable and peaked at day 28 [34]. In another study, Wall and colleagues assessed AZD1222-induced neutralizing antibodies against the SARS-CoV-2 Delta variant of concern. Two doses of AZD1222 produced neutralizing antibodies against the wild type strain in all participants (100%). Moreover, 95% and 87% individuals had a measurable neutralizing antibody against the B.1.1.7 and D614G variants, respectively [35]. Jeewandara *et al*., measured immune responses to a single dose of the AZD1222 vaccine in healthcare workers. 93.4% of participants were positive for neutralizing antibody production, regardless of gender and age. Hemagglutination tests for antibodies to the RBD were done in a sub-cohort, and ACE2 blocking antibodies were detected in 97.1% of naive people [36]. Moreover, Wall *et al.* investigated the ability of AZD1222 vaccination to elicit neutralizing antibodies against SARS-CoV-2 (Delta) in 106 participants. According to the result, 87% of individuals had measurable neutralizing antibodies against the B.1.1.7 and D614G variants, but only 62% of participants had quantifiable NAbTs against B.1.617.2 (Delta variant) following two doses of AZD1222 [37].

In a study by Logunov et al., Sputnik V vaccine efficacy was determined among adult participants. Vaccine efficacy in this study was 92%, which was very similar to our study (91%) [24]. Moreover, in a study by Claro *et al*., they assessed the antibody (IgG) response against the RBD of the spike protein and the Nucleocapsid protein (NP) in Venezuela after the vaccination by Sputnik V. Antibody responses against RBD and nucleocapsid protein were measured by ELISA. All of the participants demonstrated a strong IgG immune response against RBD after the second dose, but only 58% of participants had an immune response after the first dose [38]. In another study by Rossi and colleagues, among health care workers in Argentina, SARS-CoV-2 specific antibody responses were evaluated after vaccination by Sputnik V. IgG anti-spike titers and neutralizing capacity were determined after two doses, and 94% of participants developed spike-specific IgG antibodies. Interestingly, a single Sputnik V dose elicited higher antibody levels in previously infected individuals [39]. Also, Gushchin *et al.* evaluated the neutralizing activity of sera from Sputnik V vaccinated subjects against variants of concern, such as the alpha variant. The data obtained indicated no significant differences in virus-neutralizing activity against the alpha variant [40].

There are many studies on the effect of the Sinopharm vaccine on the creation of neutralizing antibodies against SARS-CoV-2. Holt *et al*., performed a study to evaluate the antibody responses following vaccination with the Sinopharm vaccine in the UAE after two doses (1296 participants). The antibody responses were measured 14–21 days after the second dose by means of chemiluminescence immunoassay technology, and neutralizing antibody testing was carried out by a blocking enzyme-linked immunosorbent assay. According to the result, 56% of participants had a positive anti-spike antibody against SARS-CoV-2, which was almost similar to ours (61%) [41]. In another study by Jeewandara *et al.,* the kinetics of immune responses following the Sinopharm/BBIBP-CorV was measured in Sri Lankans. SARS-CoV-2 specific total antibodies were evaluated in 83 individuals by ELISA, after the second dose. In their study, RBD specific antibodies were measured by ELISA, and about 95% of participants had measurable SARS-CoV-2 specific total antibodies [42]. Moreover, Ferenc and colleagues determined virus neutralizing antibody responses after the second dose of Sinopharm Covid-19 vaccine in 450 participants. Outcomes were examined in a multivariable model for gender and age. In a similar vein to our study, gender was slightly correlated with the antibody titers [43]. Similar to our study, gender had no significant effect on the efficacy of Sinopharm vaccine.

Various studies have been performed on the efficacy of the Covaxin vaccine. In a study by Ella, Covaxin vaccine efficacy was measured in Indian hospitals. Participants were followed two weeks after the second vaccination, and vaccine efficacy was reported at 77.8%, which was slightly more than our results (67%) [30]. In another study by Singh *et al.*, antibody response was determined after the Covaxin (BBV-152) vaccine among 515 healthcare employees in India. An anti-spike antibody titer was measured on day 21 after vaccination. The IgG to SARS-CoV-2 directed against the spike protein was assayed with an indirect chemiluminescence immunoassay (CLIA). About 44% of participants showed seropositivity after vaccination, which was almost similar to our result (70%). Also similar to our study, no difference was observed with gender [44]. In a study by Kumar, antibody responses to the BBV152 vaccine were measured in healthcare professionals. Serological testing for anti-spike antibody measurement was performed using a chemiluminescence immunoassay. According to their results, about 76% of participants showed seropositivity after vaccination, which is higher than our result (67%) [45].

Finally, in another study, Covid-19 vaccine efficacy was done by Siddique and Ahmed in Pakistan. In this study, the efficacy of various vaccines, including Sputnik V, AZD-1222, and Sinopharm vaccines, was evaluated. The results showed that the efficacy of Sputnik V, AZD-1222, and Sinopharm vaccines was 92%, 70%, and 79%, respectively, which compared to our study. However, the efficacy of AZD-1222 and Sinopharm vaccine in this study was reported to be lower and higher than our study, respectively [46].

**4. Conclusion**

The results of this study showed that vector-based vaccines have more efficacy in producing humoral immunity than inactivated vaccines, and gender does not affect the effectiveness of these vaccines. Further studies need to evaluate the duration of protection immunity acquired by COVID-19 vaccines.

***5. Methods***

***5.1 Design study***

From May to August 2021, personnel of Birjand University of Medical Sciences, including healthcare workers, students, and administrative staff, who wanted to receive the COVID-19 vaccine were invited to participate in the study. Participants donated 5 milliliters of their venous blood before receiving the first dose of vaccine and two weeks after the second dose and completed an online questionnaire. The questionnaire consisted of questions about demographic data, the history of previous COVID-19 infection, as well as the date and type of received vaccines. Sera from collected blood were separated by centrifugation and stored at -20^o^C until analysis. To evaluate the vaccine-induced humoral response against COVID-19, sera were checked for anti-RBD neutralizing antibody by PT-SARS-CoV-2-Neutralizing-Ab-96 (Pishtazteb Co., Tehran, Iran) kit, in duplicate.

X neutralizing antibody

***5.2. Calculate the immunological status ratio (ISR) of the sample***

The amount of ISR was obtained by dividing the optical density (OD) of each sample by the cut-off value. ISR was calculated according to the following formula. Considering that the experiments were performed in duplicate, to calculate the ISR, the average OD of the samples was first calculated and then divided by the cut-off value. According to the kit instructions, ISR values greater than 1.1, less than 0.8, and those values in between were considered positive, negative, and borderline, respectively. An ISR between 0.8 and 1.1 was considered borderline according to the kit instructions, and these cases were repeated. In the new experiments, results ≥1 and results <1 were considered as immunized and non-immunized, respectively.

$$IRS= \frac{OD of the clinical sample}{Cut off=0.22+OD of Negative control}$$

***5.3. Inclusion/exclusion criteria***

The inclusion criteria for this study were injections of both doses of one of the vaccines, Sputnik V, AZD-1222, Covaxin, and Sinopharm. The exclusion criteria were having a positive COVID-19 test through the study period and being unwilling to donate blood on time.

***5.4. Ethical approval***

This study was accepted on April 17, 2021 by the Ethics Committee of the Birjand University of Medical University (IR.BUMS.REC.1400.027), and all participants filled out the consent form.

***5.5. Statistical Analysis***

The data was analyzed by the SPSS software version 22.0 (SPSS Inc., Chicago, IL, USA). The Chi-square test and Student’s t-test with a significance value p < 0.05 were used.

***6. Declarations***

***Ethics approval and consent to participate:*** The project was found to be in accordance with the ethical principles and the national norms and standards for conducting medical research in Iran. This confirmation was issued by the “Research Ethics Committees of Birjand University of Medical Sciences” on 2021.04.19 (Approval ID: IR.BUMS.REC.1400.027).

***Consent for publication:*** Not applicable.

***Availability of data and materials:*** Not applicable.

***Competing interests:*** The authors declare that they have no competing interests.

***Funding:*** The project was financially supported by Birjand University of

Medical Sciences, Birjand, Iran (Grant number: 5653).

***Authors' contributions:*** HZ: Conceptualization, design of the work, interpretation of data, and drafted the work. HR: Acquisition, analysis, and interpretation of data. AF: Acquisition and analysis. SN: Design of the work. SM: Interpretation of data. SGR: Drafted the work and substantively revised it. MF: Conceptualization, design of the work, drafted the work and substantively revised it. All authors read and approved the final manuscript.

***Acknowledgements:*** Not applicable.

**References**

[1] F.-C. Zhu, Y.-H. Li, X.-H. Guan, L.-H. Hou, W.-J. Wang, J.-X. Li, S.-P. Wu, B.-S. Wang, Z. Wang, L. Wang, Safety, tolerability, and immunogenicity of a recombinant adenovirus type-5 vectored COVID-19 vaccine: a dose-escalation, open-label, non-randomised, first-in-human trial, The Lancet 395(10240) (2020) 1845-1854.

[2] F.-C. Zhu, X.-H. Guan, Y.-H. Li, J.-Y. Huang, T. Jiang, L.-H. Hou, J.-X. Li, B.-F. Yang, L. Wang, W.-J. Wang, Immunogenicity and safety of a recombinant adenovirus type-5-vectored COVID-19 vaccine in healthy adults aged 18 years or older: a randomised, double-blind, placebo-controlled, phase 2 trial, The Lancet 396(10249) (2020) 479-488.

[3] S. Matta, K.K. Chopra, V.K. Arora, Morbidity and mortality trends of Covid 19 in top 10 countries, indian journal of tuberculosis (2020).

[4] N. Zhu, D. Zhang, W. Wang, X. Li, B. Yang, J. Song, X. Zhao, B. Huang, W. Shi, R. Lu, A novel coronavirus from patients with pneumonia in China, 2019, New England journal of medicine (2020).

[5] P.M. Folegatti, K.J. Ewer, P.K. Aley, B. Angus, S. Becker, S. Belij-Rammerstorfer, D. Bellamy, S. Bibi, M. Bittaye, E.A. Clutterbuck, Safety and immunogenicity of the ChAdOx1 nCoV-19 vaccine against SARS-CoV-2: a preliminary report of a phase 1/2, single-blind, randomised controlled trial, The Lancet 396(10249) (2020) 467-478.

[6] A. Raveendran, Long COVID-19: Challenges in the diagnosis and proposed diagnostic criteria, Diabetes & Metabolic Syndrome 15(1) (2021) 145.

[7] F. Salamanna, F. Veronesi, L. Martini, M.P. Landini, M. Fini, Post-COVID-19 Syndrome: The Persistent Symptoms at the Post-viral Stage of the Disease. A Systematic Review of the Current Data, Frontiers in medicine 8 (2021) 392.

[8] A. Parkin, J. Davison, R. Tarrant, D. Ross, S. Halpin, A. Simms, R. Salman, M. Sivan, A Multidisciplinary NHS COVID-19 Service to Manage Post-COVID-19 Syndrome in the Community, Journal of Primary Care & Community Health 12 (2021) 21501327211010994.

[9] E.E. Walsh, R.W. Frenck Jr, A.R. Falsey, N. Kitchin, J. Absalon, A. Gurtman, S. Lockhart, K. Neuzil, M.J. Mulligan, R. Bailey, Safety and immunogenicity of two RNA-based Covid-19 vaccine candidates, New England Journal of Medicine 383(25) (2020) 2439-2450.

[10] F. Amanat, F. Krammer, SARS-CoV-2 vaccines: status report, Immunity 52(4) (2020) 583-589.

[11] M.J. Hossain, M.R. Kuddus, M.A. Rashid, M.Z. Sultan, Understanding and dealing the SARS-COV-2 infection: an updated concise review, Bangladesh Pharmaceutical Journal 24(1) (2021) 61-75.

[12] H. Zare, H. Rezapour, S. Mahmoodzadeh, M. Fereidouni, Prevalence of COVID-19 vaccines (Sputnik V, AZD-1222, and Covaxin) side effects among healthcare workers in Birjand city, Iran, International immunopharmacology 101 (2021) 108351.

[13] M. Mackett, G.L. Smith, B. Moss, Vaccinia virus: a selectable eukaryotic cloning and expression vector, Proceedings of the National Academy of Sciences 79(23) (1982) 7415-7419.

[14] D.A. Jackson, R.H. Symons, P. Berg, Biochemical method for inserting new genetic information into DNA of Simian Virus 40: circular SV40 DNA molecules containing lambda phage genes and the galactose operon of Escherichia coli, Proceedings of the National Academy of Sciences 69(10) (1972) 2904-2909.

[15] H. Fathizadeh, S. Afshar, M.R. Masoudi, P. Gholizadeh, M. Asgharzadeh, K. Ganbarov, Ş. Köse, M. Yousefi, H.S. Kafil, SARS-CoV-2 (Covid-19) vaccines structure, mechanisms and effectiveness: A review, International Journal of Biological Macromolecules (2021).

[16] S. Xia, K. Duan, Y. Zhang, D. Zhao, H. Zhang, Z. Xie, X. Li, C. Peng, Y. Zhang, W. Zhang, Effect of an inactivated vaccine against SARS-CoV-2 on safety and immunogenicity outcomes: interim analysis of 2 randomized clinical trials, Jama 324(10) (2020) 951-960.

[17] I. BARBERIS, P. MYLES, S. Ault, N. Bragazzi, M. Martini, History and evolution of influenza control through vaccination: from the first monovalent vaccine to universal vaccines, Journal of preventive medicine and hygiene 57(3) (2016) E115.

[18] C. Liu, L. Mendonça, Y. Yang, Y. Gao, C. Shen, J. Liu, T. Ni, B. Ju, C. Liu, X. Tang, The architecture of inactivated SARS-CoV-2 with postfusion spikes revealed by cryo-EM and cryo-ET, Structure 28(11) (2020) 1218-1224. e4.

[19] A. Kumar, T.S. Meldgaard, S. Bertholet, Novel platforms for the development of a universal influenza vaccine, Frontiers in immunology 9 (2018) 600.

[20] Q. Gao, L. Bao, H. Mao, L. Wang, K. Xu, M. Yang, Y. Li, L. Zhu, N. Wang, Z. Lv, Development of an inactivated vaccine candidate for SARS-CoV-2, Science 369(6499) (2020) 77-81.

[21] A.K. Singh, S.R. Phatak, R. Singh, K. Bhattacharjee, N.K. Singh, A. Gupta, A. Sharma, Antibody Response after Second-dose of ChAdOx1-nCOV (CovishieldTM) and BBV-152 (CovaxinTM) among Health Care Workers in India: Final Results of Cross-sectional Coronavirus Vaccine-induced Antibody Titre (COVAT) study, medRxiv (2021).

[22] G.N. Sapkal, P. Yadav, R. Ella, G. Deshpande, R. Sahay, N. Gupta, V.K. Mohan, P. Abraham, S. Panda, B. Bhargava, Neutralization of UK-variant VUI-202012/01 with COVAXIN vaccinated human serum, BioRxiv (2021).

[23] J.H. Kim, F. Marks, J.D. Clemens, Looking beyond COVID-19 vaccine phase 3 trials, Nature medicine 27(2) (2021) 205-211.

[24] D.Y. Logunov, I.V. Dolzhikova, O.V. Zubkova, A.I. Tukhvatullin, D.V. Shcheblyakov, A.S. Dzharullaeva, D.M. Grousova, A.S. Erokhova, A.V. Kovyrshina, A.G. Botikov, Safety and immunogenicity of an rAd26 and rAd5 vector-based heterologous prime-boost COVID-19 vaccine in two formulations: two open, non-randomised phase 1/2 studies from Russia, The Lancet 396(10255) (2020) 887-897.

[25] A.T. Huang, B. Garcia-Carreras, M.D. Hitchings, B. Yang, L.C. Katzelnick, S.M. Rattigan, B.A. Borgert, C.A. Moreno, B.D. Solomon, I. Rodriguez-Barraquer, A systematic review of antibody mediated immunity to coronaviruses: antibody kinetics, correlates of protection, and association of antibody responses with severity of disease, MedRxiv (2020).

[26] A.E. Muruato, C.R. Fontes-Garfias, P. Ren, M.A. Garcia-Blanco, V.D. Menachery, X. Xie, P.-Y. Shi, A high-throughput neutralizing antibody assay for COVID-19 diagnosis and vaccine evaluation, Nature communications 11(1) (2020) 1-6.

[27] X. Cao, W. Li, T. Wang, D. Ran, V. Davalos, L. Planas-Serra, A. Pujol, M. Esteller, X. Wang, H. Yu, Accelerated biological aging in COVID-19 patients, Nature communications (2022).

[28] Z.-P. Yan, M. Yang, C.-L. Lai, COVID-19 Vaccines: A Review of the Safety and Efficacy of Current Clinical Trials, Pharmaceuticals 14(5) (2021) 406.

[29] H. Wang, Y. Zhang, B. Huang, W. Deng, Y. Quan, W. Wang, W. Xu, Y. Zhao, N. Li, J. Zhang, Development of an inactivated vaccine candidate, BBIBP-CorV, with potent protection against SARS-CoV-2, Cell 182(3) (2020) 713-721. e9.

[30] R. Ella, S. Reddy, H. Jogdand, V. Sarangi, B. Ganneru, S. Prasad, D. Das, D. Raju, U. Praturi, G. Sapkal, Safety and immunogenicity of an inactivated SARS-CoV-2 vaccine, BBV152: interim results from a double-blind, randomised, multicentre, phase 2 trial, and 3-month follow-up of a double-blind, randomised phase 1 trial, The Lancet Infectious Diseases (2021).

[31] N. Ghiasi, R. Valizadeh, M. Arabsorkhi, T.S. Hoseyni, K. Esfandiari, T. Sadighpour, H.R. Jahantigh, Efficacy and side effects of Sputnik V, Sinopharm and AstraZeneca vaccines to stop COVID-19; a review and discussion, (2021).

[32] S. Siddique, S. Ahmed, COVID-19 Vaccines in Pakistan: Efficacy, Adverse Effects and Availability, Journal of Islamabad Medical & Dental College 10(2) (2021) 125-130.

[33] M. Voysey, S.A.C. Clemens, S.A. Madhi, L.Y. Weckx, P.M. Folegatti, P.K. Aley, B. Angus, V.L. Baillie, S.L. Barnabas, Q.E. Bhorat, Safety and efficacy of the ChAdOx1 nCoV-19 vaccine (AZD1222) against SARS-CoV-2: an interim analysis of four randomised controlled trials in Brazil, South Africa, and the UK, The Lancet 397(10269) (2021) 99-111.

[34] K.J. Ewer, J.R. Barrett, S. Belij-Rammerstorfer, H. Sharpe, R. Makinson, R. Morter, A. Flaxman, D. Wright, D. Bellamy, M. Bittaye, T cell and antibody responses induced by a single dose of ChAdOx1 nCoV-19 (AZD1222) vaccine in a phase 1/2 clinical trial, Nature medicine 27(2) (2021) 270-278.

[35] E.C. Wall, M. Wu, R. Harvey, G. Kelly, S. Warchal, C. Sawyer, R. Daniels, L. Adams, P. Hobson, E. Hatipoglu, AZD1222-induced neutralising antibody activity against SARS-CoV-2 Delta VOC, The Lancet 398(10296) (2021) 207-209.

[36] C. Jeewandara, A. Kamaladasa, P.D. Pushpakumara, D. Jayathilaka, I.S. Aberathna, D.R.S.R. Danasekara, D. Guruge, T. Ranasinghe, S. Dayarathna, T. Pathmanathan, Immune responses to a single dose of the AZD1222/Covishield vaccine in health care workers, Nature Communications 12(1) (2021) 1-9.

[37] E.C. Wall, M. Wu, R. Harvey, G. Kelly, S. Warchal, C. Sawyer, R. Daniels, L. Adams, P. Hobson, E. Hatipoglu, Ability of AZD1222 vaccination to elicit neutralising antibodies against SARS-CoV-2 VOC B. 1.617. 2 (Delta), Lancet (London, England) 398(10296) (2021) 207.

[38] F. Claro, D. Silva, M. Rodriguez, H.R. Rangel, J.H. de Waard, Immunoglobulin G antibody response to the Sputnik V vaccine: previous SARS-CoV-2 seropositive individuals may need just one vaccine dose, International Journal of Infectious Diseases 111 (2021) 261-266.

[39] A.H. Rossi, D.S. Ojeda, A. Varese, L. Sanchez, M.M.G.L. Ledesma, I. Mazzitelli, A.A. Juliá, S.O. Rouco, H.M. Pallarés, G.S.C. Navarro, Sputnik V vaccine elicits seroconversion and neutralizing capacity to SARS-CoV-2 after a single dose, Cell Reports Medicine 2(8) (2021) 100359.

[40] V.A. Gushchin, I.V. Dolzhikova, A.M. Shchetinin, A.S. Odintsova, A.E. Siniavin, M.A. Nikiforova, A.A. Pochtovyi, E.V. Shidlovskaya, N.A. Kuznetsova, O.A. Burgasova, Neutralizing activity of sera from Sputnik V-vaccinated people against variants of concern (VOC: B. 1.1. 7, B. 1.351, P. 1, B. 1.617. 2, B. 1.617. 3) and Moscow endemic SARS-CoV-2 variants, Vaccines 9(7) (2021) 779.

[41] S.G. Holt, S. Mahmoud, W. Ahmed, J.M. Acuna, A.K. Al Madani, I. Eltantawy, W.A. Zaher, G.J. Goodier, N.A. Al Kaabi, A.A. Al Obaidli, An analysis of antibody responses and clinical sequalae of the Sinopharm HB02 COVID19 vaccine in dialysis patients in the United Arab Emirates, Nephrology (2021).

[42] C. Jeewandara, I. Aberathna, P. Pushpakumara, A. Kamaladasa, D. Guruge, A. Wijesinghe, B. Gunasekara, S. Tanussiya, H. Kuruppu, T. Ranasinghe, Persistence of antibody and T cell responses to the Sinopharm/BBIBP-CorV vaccine in Sri Lankan individuals, Medrxiv (2021).

[43] T. Ferenci, B. Sarkadi, Virus neutralizing antibody responses after two doses of BBIBP-CorV (Sinopharm, Beijing CNBG) vaccine, medRxiv (2021).

[44] A.K. Singh, S.R. Phatak, R. Singh, K. Bhattacharjee, N.K. Singh, A. Gupta, A. Sharma, Antibody response after first and second-dose of ChAdOx1-nCOV (CovishieldTM®) and BBV-152 (CovaxinTM®) among health care workers in India: The final results of cross-sectional coronavirus vaccine-induced antibody titre (COVAT) study, Vaccine 39(44) (2021) 6492-6509.

[45] N.P. Kumar, C. Padmapriyadarsini, K.U. Devi, V. Banurekha, A. Nancy, C.G. Kumar, M.V. Murhekar, N. Gupta, S. Panda, S. Babu, Antibody responses to the BBV152 vaccine in individuals previously infected with SARS-CoV-2: A pilot study, The Indian journal of medical research 153(5-6) (2021) 671.

[46] S. Siddique, S. Ahmed, COVID-19 Vaccines in Pakistan: Efficacy, Adverse Effects and Availability, JOURNAL OF ISLAMABAD MEDICAL & DENTAL COLLEGE 10(2) (2021) 125-130.
